# Influence of Selective Conditions on Various Composite Sorbents for Enhanced Removal of Copper (II) Ions from Aqueous Environments

**DOI:** 10.3390/ijerph16234596

**Published:** 2019-11-20

**Authors:** Rebecca O. Adeeyo, Joshua N. Edokpayi, Olugbenga S. Bello, Adeyemi O. Adeeyo, John O. Odiyo

**Affiliations:** 1 School of Environmental Sciences, University of Venda, Thohoyandou Private Bag X5050, South Africa; firstrebby@gmail.com; 2 Hydrology and Water Resource Department, School of Environmental Sciences, University of Venda, Thohoyandou Private Bag X5050, South Africa; joshua.edokpayi@univen.ac.za (J.N.E.); john.odiyo@univen.ac.za (J.O.O.); 3 Department of Pure and Applied Chemistry, Faculty of Pure and Applied Sciences, P.M.B. 4000, Ladoke Akintola University of Technology, Ogbomoso 210214, Nigeria; osbello@lautech.edu.ng

**Keywords:** copper, nano-polymer adsorbent, optimum condition, polymer adsorbent, remediation

## Abstract

Numerous pollutants, including dyes, heavy metals, pesticides, and microorganisms, are found in wastewater and have great consequences when discharged onto natural freshwater sources. Heavy metals are predominantly reported in wastewater. Heavy metals are persistent, non-biodegradable and toxic, transforming from a less toxic form to more toxic forms in environmental media under favourable conditions. Among heavy metals, copper is dominantly found in wastewater effluent. In this review, the effects of high concentration of copper in plants and living tissues of both aquatic animals and humans are identified. The performance of different polymer adsorbents and the established optimum conditions to assess the resultant remediation effect as well as the amount of copper removed are presented. This procedure allows the establishment of a valid conclusion of reduced time and improved Cu (II) ion removal in association with recent nano-polymer adsorbents. Nano-polymer composites are therefore seen as good candidates for remediation of Cu ions while pH range 5–6 and room temperature were mostly reported for optimum performance. The optimum conditions reported can be applied for other metal remediation and development of potent novel adsorbents and process conditions.

## 1. Introduction

Water quality and its sustainability are essential for the survival of both human and aquatic life on Earth. The quality of water is constantly degrading due to rapid industrialisation and urbanisation This has contributed to an increase in the number of pollutants discharged into water bodies [1,2]. The existence of such water pollutants has been a threat to the entire biosphere, and their elimination or minimisation has become important. Water pollutants include dyes, heavy metals, pesticides and microorganisms which impact the ecology and humanity with diseases and problems. Among these, heavy metals with their non-biodegradable features are known to have high relative densities (greater than 5 g/mL) and atomic weights (between 63.5 and 200.6) [3,4,5].

Copper (II) ions is one of the widely spread heavy metals in the environment causing ecological and human health risk [6]. Copper metal exists in the environment in the form of copper metal (Cu^0^), cuprous ion (Cu^+^), and cupric ion (Cu^2+^), and the latter causes human health problems such as abdominal pain, nausea, renal damage, headache, severe mucosal irritation, central nervous system irritation and depression [6,7,8,9]. However, its high demand as a result of the economic importance will pose a significant increase in the concentration of copper released to the environment by 2050 [10]. Copper released will disperse into water-streams resulting in serious environmental deterioration [11]. Moreover, the release of Cu (II) into the environment is majorly through pipelines, mining, welding processes, electroplating processes, sewage treatment plants, and electrical processes [12]. This metal ion, sometimes at low concentrations, has deteriorated water bodies as well as drinking water and it is easily accumulated in bodies of animals, thereby causing a variety of diseases and disorders [13]. Due to this, the removal of copper II ions is important to reduce the concentration of copper which has been established to protect man and its environment. According to World Health Organization (WHO), the permissible limit for Cu (II) in drinking water is 2.0 mg/L while United States Environmental Protection Agency (USEPA) reported that the content of copper ions in industrial effluent should not exceed 1.3 mg/L [14,15].

This review has presented a new insight of optimum value within maximum adsorption capacities of polymer-modified adsorbent as well as emerging polymer nano-composites adsorbents at different experimental conditions (contact time, pH, temperature, initial concentration, etc.) for copper removal and their strength and future challenges are explicitly discussed.

## 2. Methodology

A desktop study of scholarly published articles was employed. The sources of search included science direct, google scholar and web of science. The search was restricted to articles written in English Language and covered the period 1997 to 2018. A review of studies reporting data on maximum adsorption conditions for both polymer and nanopolymer adsorbents for removing copper from aqueous was performed. The reported data were analysed using simple percentage analysis in Excel spreadsheet.

## 3. Occurrence of Copper in Environmental Media

**Figure 1:**
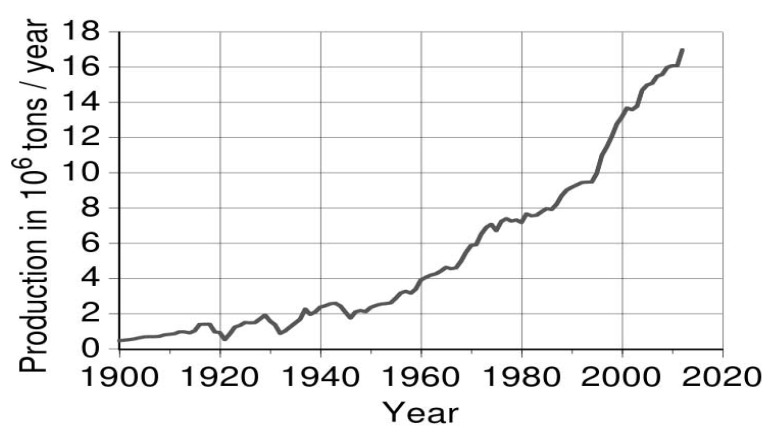
Global production of copper [15].

The unique chemical and physical properties of copper allow its extensive usage for different environmental applications such as electrical power, electronics, petrochemicals, transportation, machinery, and metallurgy. Thus, there is a great interest in the global production of copper (Figure 1) which sums up to 12 million tons per year with reserves around 300 million tons [15].

The economic activities of humans such as copper production and usage as well as its compound result in the different copper distribution in various environmental media. Table 1 summarises the copper concentration in various environmental media. Copper is known to be a naturally occurring element that is existing in the earth, oceans, lakes, and rivers [16].

Sources can be accessed via native geology, hydrogeology as well as geochemical features of the aquifer [16,18]. Additionally, the rudimentary foundation of copper is polluting the water resources through weathering of sedimentary rocks such as limestone, dolomite, shale, and sandstone. Also, some minerals or ores such as cuprite, malachite and azurite on dissolution increase the concentration of copper in the environment [19,20,21].

Moreover, wind-blown dust, volcanoes, forest fire, sea spray, decaying vegetation, urban runoffs, aerosol particles, and soil erosion are also other natural sources of copper [22]. Hazardous impacts released from volcanic eruptions into the environment have been reported to affect the climate and health of exposed individuals [23].

The major anthropogenic sources of copper are industrial, domestic and agricultural activities. High concentrations of copper from industrial activities such as painting, metal works, mining operations, refining processes, batteries and electronic manufacturing, textile as well as nuclear power, are often deposited into wastewater stream, although atmospheric deposition is also possible [18]. For instance, copper concentration in wastewater from the metal finishing industry can be extreme up to a concentration of about 10,000 mg/L [18]. The annual industrial copper discharges into freshwater is estimated as 1.4 × 10^10^ g/year, as well as the amounts of copper in sewage sludge and industrial waste that have been dumped into the ocean as 1.7 × 10^10^ g/year globally [24].

Sewage sludge application on land is a major source of copper into agricultural soil [25,26,27,28]. Moreover, agricultural activities such as the application of fertilizers on farmland, fungicidal spraying, and the use of animal wastes can lead to water pollution through copper deposits [29]. Contamination in agricultural fields by copper ions also depends on the use of various types of pesticides [30,31].

Another anthropogenic source of copper is leachate from municipal landfills and domestic wastewater. Copper concentration in leachates varies depending on the age of the landfill and the kind of waste that is deposited including the socioeconomic status of the people the landfill is serving. The concentration of copper in leachate from municipal landfills have been established to range from 0.005 to 1110 mg/L [32]. Wastewater effluents are most probably the largest contributor of the high concentration of Cu found in different water bodies, which could be from mechanically treated or untreated wastewater supplies from the filters of biological treatment plants, and waste substances from sewage outfall that is discharged into water bodies such as sea [33,34].

## 4. Toxicological Effects of Copper II Ion

**Figure 2:**
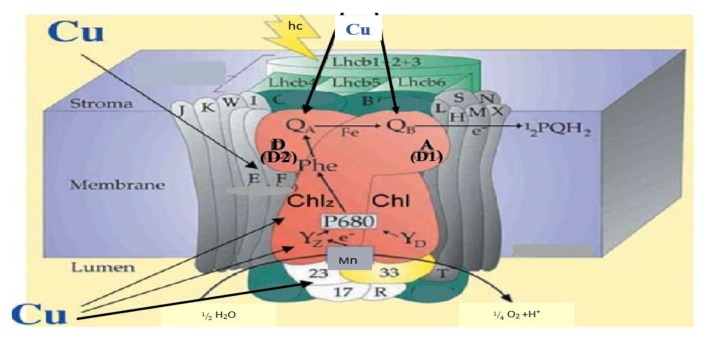
Scheme of copper action sites in phyto system II of plants [36].

Though copper is significant to man and the ecosystem, its absence could lead to serious limitations to the functioning of the living cell. Moreover, levels above 3 mg/L can negatively impact plants, aquatic biota and human health [24]. One of the essential micronutrients for plant growth is copper, because of its excellent significance in the production of seed, disease resistance, as well as other essential nutrients depending on the solubility of copper in the soil [35]. High concentrations of copper can lead to biochemical alterations, interference of several physiological and cellular processes, which potentially inhibit plant growth, photosynthesis, and respiration. The mechanism of Cu toxicity on photosynthetic electron transport established photosystem II in plants to be a sensitive site to a high concentration of copper [36]. Figure 2 shows the scheme of copper action sites in phytosystem II of plants. Consequently, this results in performance reduction, delay in growth of the root and leaf, as well as ultra-structural and anatomical alterations which frequently result in the formation and accumulation of reactive oxygen species (ROS) [37]. Besides, the growth of plants in the presence of a high concentration of copper reveals reduced biomass and chlorotic symptoms [36].

Copper ions affect the environment by inducing damage to aquatic biota and affecting the osmo-regulatory process of freshwater animals. Copper toxicity can be a short or a long-term effect, which may result in a reduction in growth, immune response, reproduction and survival of the aquatic animals. Copper is toxic to some cultured species of fishes such as catfishes and salmonids above specific concentrations [38]. For example, acute toxicity of copper sulphate was compared in tilapia (*Orechromis niloticus*) and catfish (*Clarias gariepinus*) species using the toxicity index of 96 h LC50. The author reported that copper is more toxic to *Orechromis niloticus* than *Clarias gariepinus* with their 96 h LC50 values to be 58.837 mg/L and 70.135 mg/L respectively [39,40]. The adverse effects have been demonstrated on various fish receptors like gills, olfactory receptors, and lateral line cilia as well as fish DNA [41,42].

Excessive intake of copper in humans can prompt symptoms such as abdominal pain, nausea, vomiting, headache, damages to renal tubules, respiratory difficulties, hemolysis, memory deficit, vascular collapse, hepatic necrosis, gastrointestinal bleeding, liver and kidney failure, and death [43,44]. Copper may also cause itching, dermatitis, keratinisation of the hands and sole of the feet, due to its toxicity and widespread presence in the industrial applications such as electrical, electro-plating, metal-finishing and paint industries [18,22]. As a result of this, several regulatory bodies (e.g., USEPA) came up with standards for regulating copper discharge into the environment. Table 2 summarises the maximum permissible concentration for copper.

## 5. Conventional Methods of Removing Cu II ions

Several methods including chemical precipitation, membrane filtration, electrodeposition, ion exchange, adsorption, and membrane separation have been used to remove copper (II) ions from aqueous solution with notable advantages such as simplicity of operation, high efficiency, low energy requirement, and low driving force [46]. Conversely, some inherent limitations have been discovered using these technologies.

These limitations include increased capital and maintenance cost, expensive equipment, great sensitivity to operational conditions, increase in consumption of energy, removal of metal incompletely, generation of toxic sludge and some are ineffective at low concentrations [47]. Table 3 summarises the advantages and disadvantages of the physicochemical methods of removing copper ions from wastewater. Moreover, amidst the conventional methods, adsorption is observed as the most favourable, because of its clean and fast operation, high productivity, simplicity, design, reduced cost and accessibility of diverse adsorbents [53,54,55,56].

Several substances have been studied and established for the adsorption of copper ions from wastewater. Such adsorbents have been derived from natural materials including agricultural and industrial solids wastes, montmorillonite and kaolinite, chitosan and polymeric materials [55,56,57,58].

Table 4 presents the maximum capacity of various natural adsorbents for the removal of copper in aqueous solution. Recently, studies on numerous solid-phase adsorbents such as carbon nanotubes, ion imprinted polymers, biosorbents, and nanoparticles that serve as alternatives have been established. These alternative sorbents are efficient and have a high performance during the removal of their targeted metal [59,60,61,62].

## 6. Polymer-Based Adsorbents

Polymer adsorbents’ properties include adjustable surface chemistry, vast surface area, pore size distribution, seamless mechanical strength and they are very easy to regenerate [79,80,81,82,83]. This makes polymer adsorbents excellent materials for the removal of Cu (II) from water streams.

Polymer adsorbents can be classified into biopolymers and synthetic polymers. Biopolymers such as chitin and its derivatives, cellulose, alginate, carrageenan, lignin, proteins, chitosan and polysaccharides are from renewable resources which are biodegradable, non-toxic and have an excellent capability to mix with a variety of molecules by physical and chemical interactions [84]. The presence of hydroxyl, amine, amide, and carboxyl functional group makes it an equally excellent adsorbent. An investigation on the removal of copper (II) ions by chitosan solution via homogenous adsorption resulted in adsorption capacity of 405 mg/g [85].

**Figure 3:**
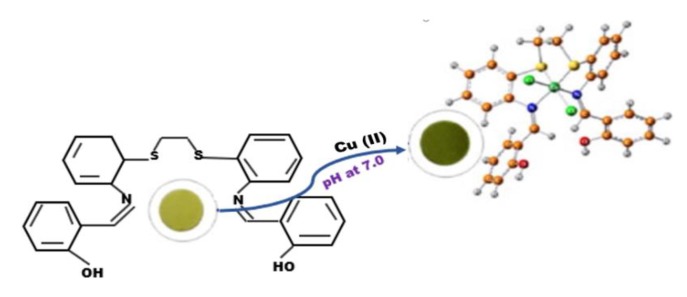
Efficient meso-adsorbent prepared for trace Cu (II) detection and removal [9].

Synthetic polymers have been reported to perform excellently during the sequestration of copper ion in aqueous solution when functionalised with amino or carboxylic acid groups for specific interaction. For example, Figure 3 presents a synthetic meso-adsorbent prepared of trace discovery and adsorption of Cu (II) ions at pH 7. These synthetic polymers enhanced the adsorption capacity with a direct association with the chelating groups in the polymer structure [80]. Samadi et al. [86] studied the removal of Cu (II) ions using polymer derivatives of polystyrene-alt-maleic anhydride from aqueous solution. Table 5 presents the optimised conditions using polymer for Cu (II) sequestration.

## 7. Polymer Nano-Composite Based Adsorbent

**Figure 4:**
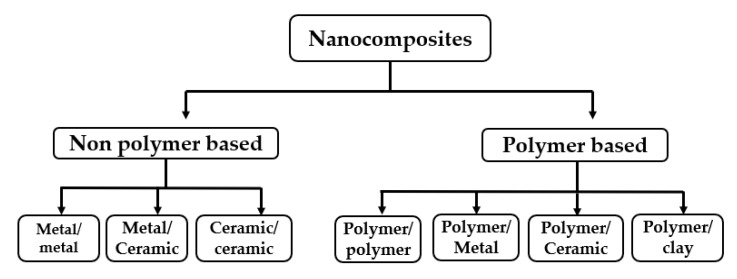
Classification of nano-composite [99].

Nanocomposites are multi-phasic materials, in which at least one of the phases shows its dimension in the nano range (1–100 nm). Nano-composite materials have currently emerged as substitutes to overcome different limitations in engineering materials and present a high adsorption capacity, granulometric properties, chemical, and thermal stabilities, reproducibility, with better selectivity for the copper ions removal compared to pure organic and inorganic materials [97]. Conversely, they are too small to be used directly because of their large specific surface energies. The combination of nanoparticles with polymer material creates a specific property that enhances the adsorption of copper ions. Figure 4 shows the classification of nanocomposites as well as its combining nanoparticles. Nanocomposites are enhanced with either polymeric or non-polymeric material according to their dispersed matrix. The matrix from polymer material has been proved to be ideal support for the fabrication of composites as adsorbents, considering the adjustable surface functionality and the excellent mechanical strength [98].

Therefore, the synthesised polymer nano-composite adsorbents display some unique properties like easy preparation, cost-effectiveness, dimensional ability, activated functionality, environmental stability, effective binding sites along the walls of the polymers with large surface area, and pore volume, thus making it a significant area of current research and development [100].

### Techniques for Preparing Polymer Nano-Composite Adsorbent for Copper (II) Removal

Many methods have been developed to synthesise composites of polymers and nanomaterials. To obtain the expected composite functionality, the development is done according to their ‘preparation path’. The methods of synthesis include direct compounding and in-situ synthesis.

The direct compounding method involves the synthesis of nanomaterials and polymers before blending using different methods. Direct compounding is an excellent method of preparing polymer nanocomposites due to advantages such as its fitness for large scale production and lower cost. The major limitation of this method is that nanoparticles have a high tendency to form aggregates that delay homogenous dispersion of nanoparticles in polymeric matrices. This overcomes the need for addition of dispersants or compatibilisers; application of different surface modifications/chemical treatments to nanomaterial or polymers and optimisation of synthesised parameters such as temperature, shear force, time, mixing speed, and configuration of the reactor [101,102]. In direct compounding, techniques of synthesis involve (i) solution intercalation (ii) sol gel method (iii) electro spinning (iv) self-assembly (v) melting intercalation.

Among these polymer synthesis nano-techniques, electrospinning and in-situ techniques have been efficiently used for the removal of copper (II) in aqueous solution. The electro-spinning method has three parts as a high-voltage supplier which is used to acquire an electrically charged jet of a composite solution in the needle. The charged jet is ejected from the tip of the needle, completely and the solvent is vaporised, which leads to the formation of nanocomposite on the collector [103,104]. The advantages of electrospinning include simplicity, low cost, high speed, vast material selection, and versatility [105]. An example of an electrospinning application for the removal of copper (II) ions in aqueous solution using polyethylene oxide/chitosan nanofiber membrane has been previously described by Aliabadi et al. [106] who concluded that the removal of copper (II) ion is feasible, spontaneous and endothermic.

**Figure 5:**
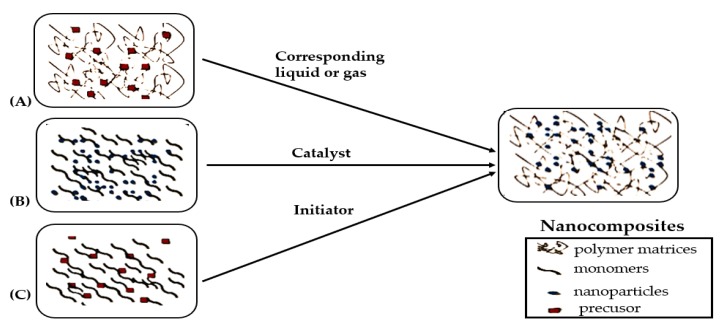
In situ polymerisation method. (**A**) the polymer was blended with metal ions as starting material, (**B**) Nanomaterial and the monomer were used as starting material, (**C**) Preparation of nanoparticles and polymer simultaneously [93].

In situ- polymerisation is the swelling of the filler in monomer solution as the low-molecular-weight monomer seeps amid the interlayers causing the swelling [107]. The use of heat, radiation, initiator diffusion by organic initiator or catalyst fixed through cationic exchange starts the polymerisation process [108]. Intercalated or exfoliated nanocomposites are formed as the monomer polymerises in between the interlayers. The advantage of this technique is the simplicity, effectiveness, and prevention of particle agglomeration while maintaining a good spatial distribution in the polymer matrix. Figure 5 presents the synthesis involved during in-situ polymerisation. Polypyrole nanocomposite (ppy/TiO_2_) was prepared by in situ polymerisation for the removal of copper (II) ions and was found to be effective within the equilibrium time of 30 min [109]. Table 6 summarises the methods of preparation of nano-polymer adsorbents and their maximum adsorption conditions for the removal of copper (II) ions.

## 8. Result and Discussion

**Figure 6:**
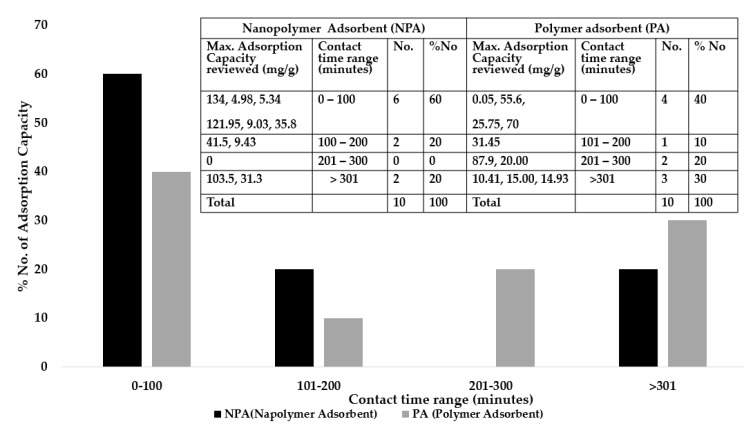
Contact time with maximum adsorption capacities for polymer and nanopolymer adsorbents.

**Figure 7:**
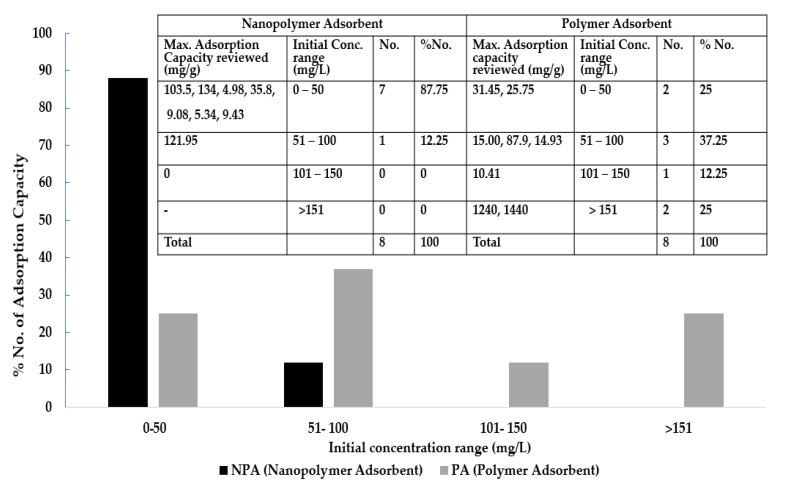
Concentrations with adsorption capacities for polymer and nanopolymer adsorbents.

**Figure 8:**
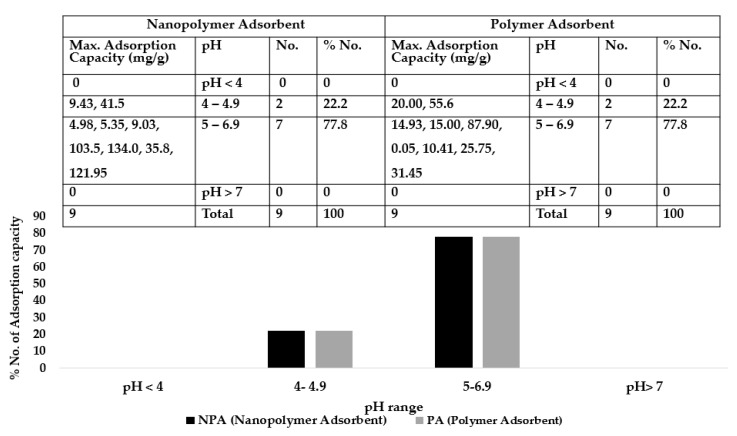
pH at maximum adsorption capacities for polymer and nanopolymer adsorbent.

Factors influencing the adsorption of copper (II) ions are optimum contact time, pH and initial concentration. Figure 6, Figure 7 and Figure 8 give the number of maximum adsorption capacity reviewed against contact time, initial concentration and pH.

### 8.1. Optimum Contact Time

Figure 6 presents optimum contact time for adsorption capacities for polymer and nanopolymer adsorbents. Contact time for nanopolymer adsorbents at maximum adsorption capacities of 134, 4.98, 5.34, 9.03, 35.8 and 121.95 mg/g occur within 0–100 min contributing 60% of the total adsorption capacity reviewed. Optimum adsorption capacities of 0.05, 25.75, 55.6 and 70 mg/g were recorded for polymer adsorbents within 0–100 min and were found to be 40% of the total adsorption capacity reviewed. There is a notable trend of a decrease in maximum sorption for equilibrium time in nanopolymer adsorbent. The variation in maximum adsorption in the studied materials indicates that material composition also affects maximum adsorption with enhanced optimum sorption processes favoured within the shortest time limit considered (optimum time for adsorption) in nanopolymer composite than in polymer adsorbent. Moreover, various kinetic models such as pseudo-first order, pseudo-second order, intraparticle diffusion, and Elovich’s equation for adsorption efficiency were studied to describe the adsorption processes of Cu (II) and explain the mechanism involved based on the concentration of the solution (mostly 10 mg/L). Pseudo-second order kinetic model described the whole adsorption processes well as chemisorption in nature as the limiting rate step for all the adsorption capacities [120,121].

The short sorption time for nano-polymer adsorbents may be due to the availability of an uncovered surface and active sites in the nanocomposite adsorbent. Heiba et al. [103] revealed a short sorption time of 40 min in the removal of copper II ions using CMC/MMT nanocomposites because vacant binding sites are easily accessible on nanocomposite, which results to further reactivity of these active sites and covered with Cu^2+^ ions, therefore, no additional binding of Cu^2+^. Moreover, the percentage rate of copper removal is higher at the commencement of the process to achieve short optimum contact time due to large surface area and pore size of the adsorbent being available for the adsorption process [122]. The shorter equilibrium time means a shorter agitation period (less energy consumption) which offers an economic advantage for the scale application and therefore, result in cost reduction.

### 8.2. Optimum Initial Concentration

Figure 7 shows the maximum adsorption capacities of copper for initial metal concentration for both polymer and nanopolymer adsorbent. Maximum adsorption capacities reported for nanopolymer adsorbent include 103.5, 134, 4.98, 35.8, 9.03, 5.34 and 9.43 mg/g constituting 87.75% of total maximum adsorption reported and was observed at initial concentration ranging from 0–50 mg/L while polymer adsorbents constituted 25% of the reported results at adsorption capacities of 31.45 mg/g and 25.75 mg/g.

The observed pattern revealed increased initial concentration, a decrease in number and percentage of maximum adsorption capacity occurs for nanopolymer adsorbent (NPA) while the number and percentage maximum adsorption capacities for polymer adsorbents (PA) are inconsistent. These results indicate that initial concentration significantly influences the uptake of copper ion and maximum adsorption in aqueous solution at different optimum initial concentrations.

It was observed that a high adsorption efficiency is likely at low initial concentration for nanopolymer composite adsorbents. Cai et al. [114] explains the reason to be the difference in the concentration gradient between Cu^2+^ in the initial solution and its absence on the nano-adsorbent which is acting as a driving force, till all the active sorption places are taken, while adsorption process is efficient at high initial concentration of the adsorbate for polymer adsorbents. An increase in the adsorbent mass (optimum being 0.5 g) increases the number of active adsorption sites and adsorption capacity [123]. Moreover, the initial concentration in the removal of copper offers a significant driving force that overcomes all mass transfer resistances of the copper ion between the solid phase and the solution [124].

### 8.3. Optimum pH

Figure 8 presents optimum pH reported at different maximum adsorption capacities for nanopolymer and polymer adsorbents. Optimum pH values for both nano-polymer and polymer adsorbents occur at pH 5–6.9. The pH of 5–6.9 accounted for about 77.8% in both adsorbents studied which are the maximum. Nano-polymer optimally favoured adsorption of Cu II ion at pH 5–5.9 while polymer adsorbent recorded optimum function at pH of 6–6.9. At low pH (very acidic) and above pH 7, reduction of sorption capacities occurs for both materials under study. Variation in optimal pH may indicate different suitability and function in Cu II ion sorption in aqueous solutions for both materials under consideration.

The results indicate pH as an important parameter that influences the uptake of copper (II) ions because it determines the degree of ionisation, adsorbent surface charge and the speciation of the adsorbate [124]. When pH is low (pH < 4), the acidity of the solution is high, because of an increase in positive charge density and high electrostatic repulsion, which results in lesser uptake of copper ions carrying a positive charge. Thus, there is a decrease in adsorption and reduction in the number of negatively charged sites accessible for copper ions to bind due to protonation of the active sites. Also, there is competition between hydrogen ions and Cu (II), which decreases the adsorption capacity. At higher pH, copper ion is free to bind since the active sites have deprotonated, thus the competition between copper ions and protons is reduced.

Cai et al. [114] explain that precipitation of Cu (II) occurs in form of Cu(OH)_2_ due to the increasing concentration of OH^−^ ions resulting to the creation of anionic complexes of hydroxide that the maximum adsorption capacity is at 6 and the adsorption decreases by raising or lowering the pH [125]. Therefore, the concentration of the metal ions that dissolved and their adsorption on the active sites would decrease. Plohl et al. [126] reported that the uptake of Cu^2+^ most likely occurs through the deprotonated primary group (functional group). Also, for the removal of copper using silica magnetic nanocomposite, Cu^2+^ from copper hydroxide precipitates at pH 6 where the nanocomposite adsorbent is accessible due to electron donor pairing with favourable Cu^2+^ chelation. The reduction lowers the electrostatic repulsion between the copper ions and the adsorbent surface, which leads to an increase in the uptake of metal ions [127]. Several studies have reported a pH of 6 as the maximum adsorption efficiency for Cu (II) ions [94,128,129,130]. This review has established optimum pH can range from 5–6.9 for the adsorbent.

Generally, other cations such as Na^+^, K^+,^ Mg^2+^, and Ca^2+^ can be detected in several wastewaters. The existence of these cations results in high ionic strength, which invariably affects the adsorption behaviour. The effect of ionic strength on copper adsorption with these nanopolymer adsorbents were studied using salts such as NaCl, KCl, MgCl_2_, and CaCl_2_ in aqueous solution at ionic medium ranging from 0.01 and 0.1mol/L [114,131].

## 9. Conclusions

The effective adsorption capacity of nano-polymer adsorbents for copper (II) ion removal may be credited to the outstanding characteristics of nano-sized materials as well as the functional group of the synthesised polymer material for the development of novel composite materials that have high surface-active sites and increased specific surface area to volume ratio. Thus, the use of nano-polymer-based adsorbents will provide high adsorption capacities in the purification of copper ions from aqueous solutions. Other factors affecting the adsorption of the copper ion on nano-polymer adsorbents with increasing adsorption capacities are short optimum contact times, agitation, low initial concentration and circumneutral value of pH at pseudo-second order kinetic model and ambient temperature. Nano-polymer composite-based adsorbents at this experimental condition can, therefore, be recommended and used for the development of effective bioprocesses and sequestration of copper ion from aqueous solution in further studies.

## 10. Future Researches

Although there is great significance in the adsorption conditions contributing to the efficient removal of copper ions using the nano-polymer composite, some gaps still need to be filled to overcome future challenges in this line of research. The re-use and regeneration of the adsorbent material should be studied to support the life cycle impact and encourage sustainability. Moreover, two methods (electrospinning and in situ polymerisation) of synthesis are commonly used, but other techniques of synthesis such as sol-gel method, solution intercalation, melting intercalation and self-assembly should be explored for selective copper removal and removal of other heavy metals from aqueous solutions to reduce the use of solvent and increase the compatibility with industrial processes. The reduction in the use of chemicals and solvents will contribute to the manufacturing of environmentally friendly products, and the sustainability of the environment. Also, industrial treatments to remove heavy metals from aqueous solutions using nano-polymers should be studied considering the influence of the adsorption conditions with little or no modification to encourage cost effectiveness, profitability, and easy engineering application.

## Figures and Tables

**Table 1:** Concentration of Copper in natural environmental media [17].

Environmental Media		Concentration	Unit
Soil	Total content in soil	2–100	µg/g
	Soluble content in soil	<1	µg/g
Atmosphere	Aerosol	1 × 10^−7^–3.82 × 10^−4^	µg/L
Hydrosphere	Fresh water	8 × 10^−5^	µg/L
	Sea water	0.01–2.8	µg/L
Biota	Plant	1–110	µg/g
	Animal	2.4	µg/g

**Table 2:** USEPA Permissible Concentration (PC) for copper in water [45].

Element	Copper (mg/L)
PC in water	0.1
PC in wastewater discharge into the public sewage	1.0
PC in wastewater discharge into surface water	0.1

**Table 3:** Advantages and drawbacks of conventional methods for copper ions sequestration from aqueous solution [5,47,48].

Methods	Advantages	Disadvantages	Reference
Ion exchange	Treatment even at low concentration, fast kinetics	Expensive, interference of composite ion and regeneration	[49]
Coagulation-Flocculation and Sedimentation (CFS)	Simplicity and low cost.	Low density with bulky sludge	[47]
Membrane Technology	High efficiency and small footprint	Increased energy, intense disposal and maintenance difficulty	[47]
Electrolysis	Ease of operation, No requirement for chemical use	Expensive	[47]
Chemical Precipitation	High percentage removal, simplicity of operation	Bulky hydroxide and colloidal particles, Expensive	[47,50]
Membrane Filtration	High efficiency, low energy requirement, a small space due to high packing density, low driving force	High operational cost due to membrane fouling	[50]
Electrodialysis	Treatment of highly concentrated wastewater, high separation selectivity	Membrane replacement and corrosion process, high energy consumption	[51]
Microbial treatment	Ecofriendly	Scaling up, slow, difficult to standardise	[45]
Adsorption	High capacity, fast operation, simple, high metal binding	Low selectivity, regeneration is expensive	[45,52]

**Table 4:** Maximum adsorption capacity of different natural adsorbents for the removal of copper in aqueous solution.

Type of Adsorbent	Maximum Adsorption Capacity (mg/g)	References
**Agricultural waste**		
Dried sugar beet pulp	28.5	[63]
Wheatshell	8.26	[64]
Rice husk modified with NaOH	10.9	[65]
Moss	11.2	[66]
Peanut husk	10.15	[67]
Mango	42.60	[68]
Soyabean hull	154.9	[69]
Carrot Residue	32.74	[70]
**Chitosan**		
Chitosan-g-maleic acid	312.4	[71]
Cross linked Magnetic Chitosan	78.13	[72]
Chitosan	150	[73]
**Montmorillonite**		
Powdered Limestone	0.29	[74]
Anuvilia Soil	0.63	[75]
**Industrial solid waste**		
Olive oil waste	16	[76]
Saw Dust fir tree	12	[77]
Tea industry waste	8.64	[78]

Bold text indicates broad category of adsorbent sub-types.

**Table 5:** Summarised maximum adsorption conditions and their functional group for various natural and synthetically modified polymer.

Adsorbent	Functional Group	Adsorption Capacity (mg/g)	Contact Time (min)	pH	Temp (K)	Initial Conc. (mg/L)	Reference
Amine functionalized silica magnetite	-NH_2_	10.41	1440	6.5	298	150	[87]
Chitin biopolymer	-NH_2_	13–15	480	5	298	100	[22]
Grafted cassava starch with 5-chloromethyl-8-hydroxyquinoline (CMQ)	-OH	25.75	90	6	-	50	[88]
Polyamine-immobilised trimethylaniline	-C=O	1.47	-	5	-	-	[89]
Chitosan coated with polyvinyl chloride	-NH_2_, -OH	87.9	210	5		100	[90]
(*E*)-2-[(1*H*-Imidazolyl) methylene]-hydrazinecarbo thioamide ligand (EIMH)	-NH_2_	0.05	20	6	-	-	[91]
Modified acrylic acid grafted polyethylene terephthalate (PET) film	-OH	55.6	60	4	298	2000	[92]
Modified Lignin from pulping waste	-COO-	20	240	4	330	-	[93]
Polyhydroxyethylmethacrylate (PHEMA-HEMA)	-	31.45	120	6	330	10	[94]
Pristine zeolite	-	14.95	1240	55	-	100	[95]
Regenerated cellulose	-	70	30	7	-	300	[96]

**Table 6:** Summarised methods of preparation of nanopolymer adsorbent and their maximum adsorption conditions for the removal of copper (II) ions.

Nano Materials	Polymer Materials	Method of Preparation	Adsorbent	pH	Contact Time (min)	Temp (K)	Initial Conc (mg/L)	Adsorption Capacity (mg/g)	Reference
Keratin	Polyamide 6	Electrospinning	Keratin/PA6	5.8	1240	-	35	103.5	[110]
Chitosan	Polystyrene	Electrospinning	Polystyrene chitosan rectories	5.5	15	293	50	134	[111]
Fibres	Fe_2_O_3_-Al_2_O_3_	Electrospinning	Electrospun/Fe_2_O_3_	5.5	60	298	30	4.98	[112]
CMC	Montmorillonite	Electrospinning	CMC/MMTNC	5	40	-	5	5.34	[113]
Nano Fibers	Polyindole	Electrospinning	Electrospun Polyindole	6	15	293	100	121.95	[114]
MCM-41	PMMA	In-situ Polymerization	MCM-41/PMMA	4	140	298	10	41.5	[115]
Silica Kit 6	PMMA	In-situ Polymerization	PMMA/SilicaKit6	5.5	90	293	10	9.03	[116]
Amine Modified MCM-41	Nylon 6	In situ Polymerization	Amine-modified MCM-41/nylon 6	6	75	293	50	35.8	[117]
Thiol Boehmite	PMMA	In situ Polymerization	Boehmite/PMMA	4	20	-	10	9.43	[118]
Nano Fibres	Polyacrylonitrile	Electrospinning	Hydrolysed Electrospun Polyarylonitrile	5.0	300	-	-	31.3	[119]

PMMA: Polymethylmethacrylate; CMC/MMTNC: Carboxyl methylcellulose/montmorillonite nanocomposite; PA6: Polyamide 6; MCM 41: mesoporous silica 41; Fe_2_O_3_-Al_2_O_3_: Iron III Oxide-Aluminum oxide.
